# The NEIL1 G83D germline DNA glycosylase variant induces genomic instability and cellular transformation

**DOI:** 10.18632/oncotarget.20716

**Published:** 2017-09-08

**Authors:** Heather A. Galick, Carolyn G. Marsden, Scott Kathe, Julie A. Dragon, Lindsay Volk, Antonia A. Nemec, Susan S. Wallace, Aishwarya Prakash, Sylvie Doublié, Joann B. Sweasy

**Affiliations:** ^1^ Department of Microbiology and Molecular Genetics, The Markey Center for Molecular Genetics, University of Vermont, Burlington, VT, 05405, USA; ^2^ Department of Therapeutic Radiology, Yale University School of Medicine, New Haven, CT, 06510, USA; ^3^ Present address: University of New Mexico, Health Sciences Center, Albuquerque, NM, 87131, USA; ^4^ Department of Biomedical Sciences, Florida State University, Tallahassee, FL, 32306, USA; ^5^ Present address: University of South Alabama, Mitchell Cancer Institute, Mobile, AL, 36604, USA

**Keywords:** base excision repair, genomic instability, replication fork collapse, DNA glycosylase, mutagenesis

## Abstract

Base excision repair (BER) is a key genome maintenance pathway. The NEIL1 DNA glycosylase recognizes oxidized bases, and likely removes damage in advance of the replication fork. The rs5745906 SNP of the *NEIL1* gene is a rare human germline variant that encodes the NEIL1 G83D protein, which is devoid of DNA glycosylase activity. Here we show that expression of G83D NEIL1 in MCF10A immortalized but non-transformed mammary epithelial cells leads to replication fork stress. Upon treatment with hydrogen peroxide, we observe increased levels of stalled replication forks in cells expressing G83D NEIL1 versus cells expressing the wild-type (WT) protein. Double-strand breaks (DSBs) arise in G83D-expressing cells during the S and G2/M phases of the cell cycle. Interestingly, these breaks result in genomic instability in the form of high levels of chromosomal aberrations and micronuclei. Cells expressing G83D also grow in an anchorage independent manner, suggesting that the genomic instability results in a carcinogenic phenotype. Our results are consistent with the idea that an inability to remove oxidative damage in an efficient manner at the replication fork leads to genomic instability and mutagenesis. We suggest that individuals who harbor the G83D NEIL1 variant face an increased risk for human cancer.

## INTRODUCTION

Base excision repair is critically important for maintaining genomic stability because it repairs at least 20,000-50,000 lesions per cell per day that arise from the inherent instability of DNA and the presence of reactive oxygen and nitrogen species (RONs) (for a review see [1]). BER is initiated by DNA glycosylases that recognize and remove specific types of oxidized and alkylated bases in DNA (for a review see [2]). Apurinic/apyriminidinic endonuclease I (APE I) recognizes and incises the abasic site generated by a monofunctional DNA glycosylase, leaving a single nucleotide gap with a 3’OH and a 5’-deoxyribose phosphate (dRP) group. This gap is filled in by DNA polymerase beta (pol ß), which also removes the 5’dRP, and DNA ligase 3α (LIG3α) in complex with X-Ray Cross-Complimenting 1 (XRCC1) seals the nick (for a review see [3]). Bifunctional glycosylases, which usually recognize oxidative lesions, generate an abasic site and their associated lyase activity cleaves the DNA backbone via ß-elimination to generate a 3’dRP and a 5’phosphate. APE1 then catalyzes removal of the 3’dRP, leaving a 3’OH. Pol ß binds to this substrate and fills in the resulting single nucleotide gap. In both cases, the XRCC1/Ligase IIIɑ or XRCC1/Ligase I complex catalyzes ligation of the resulting ends. An alternative BER pathway, that does not depend on APE1, is utilized when the NEIL glycosylases initiate repair [4]. NEIL1 and NEIL2 catalyze excision of the damaged base via ß,δ elimination, leaving a 3’phosphate and a 5’phosphate. The 3’phosphate is removed by polynucleotide kinase, leaving a gap that is most often filled by pol ß, followed by ligation. Although NEIL3 exhibits weak lyase activity in the form of β-elimination, the base excision reaction is significantly more efficient than its lyase activity, suggesting that it functions predominantly as a monfunctional enzyme [5, 6].

The NEIL1 DNA glycosylase is a bifunctional enzyme that recognizes oxidized pyrimidines and formamidopyrimidines, and appears to excise lesions ahead of the replication fork [4, 7–12]. The rs5745906 SNP of the *NEIL1* gene has been identified predominantly in Europeans and African Americans, has a minor allele frequency of < 0.01, and is a G to A base substitution that results in a Gly83 to Asp83 (G83D) mutation. This variant was identified in patients with primary sclerosing cholangitis and cholangiocarcinoma [13]. We have previously shown that unlike WT NEIL1, the G83D variant is not able to excise oxidized bases efficiently due to a shift in the void-filling Met81 residue that stabilizes the DNA duplex once a damaged base has been extruded into the glycosylase substrate binding pocket [14]. Not surprisingly, the G83D variant is an inactive DNA glycosylase on most oxidized bases with the exception of spiroiminodihydantoin (Sp) and guanidinohydantoin (Gh), which can form stable extrahelical structures [14–17]. However, the recombinant NEIL1 G83D DNA glycosylase appears to be properly folded as it retains its lyase activity and is able to bind to substrates containing a thymine glycol (Tg):A base-pair [14, 15]. It has also been shown that NEIL1 G83D exhibits a shorter retention time at laser-induced oxidative damage, but an increased retention time at psoralen crosslinks compared to WT NEIL1 [18].

Given the role of oxidative DNA damage repair in genome maintenance and the hypothesis that BER is a tumor suppressor mechanism [19], we wished to determine whether the G83D NEIL1 variant induces an oncogenic phenotype in cells. We found that expression of G83D in MCF10A immortal human breast epithelial cells induces replication stress, resulting in genomic instability and cellular transformation. These phenotypes likely arise due to an inability of G83D to remove oxidative DNA damage at the replication fork. Our results are consistent with the idea that individuals who harbor the G83D NEIL1 variant are at increased risk for cancer.

## RESULTS

### NEIL1 G83D acts in a dominant manner *in vitro*

**Figure 1:**
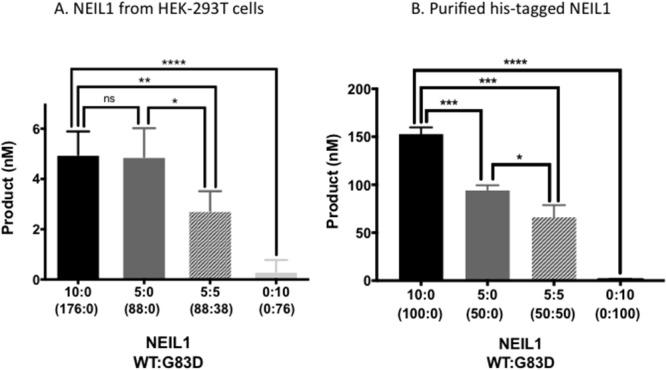
G83D is dominant to WT NEIL1 *in vitro* **(A)** The WT and G83D NEIL1 DNA glycosylases were expressed in and purified from HEK293T cells as described in Methods. Glycosylase reactions were performed in a total volume of 20 μL, as described in Methods, using only WT, G83D, or mixtures of WT and G83D as indicated by the enzyme ratios on the X-axis of the graph. These ratios are expressed in volume (μl). Subsequent quantitative western blotting, an example of which is shown in Supplementary Figure 1, revealed that 10:0 WT:G83D is 176 nM WT:0 nM G83D; 5:0 WT : G83D is 88 nM WT to 0 nM G83D; 5:5 WT : G83D is 88 nM WT : 37.5 nM G83D; and 0:10 WT:G83D is 0 nM WT to 75 nM G83D. * p ≤ 0.05, ** p ≤ 0.01, *** p ≤ 0.001, unpaired t-test. **(B)** Histidine-tagged (his-tagged) NEIL1-WT and G83D were purified from *E. coli* as described [14]. Glycosylase reactions with purified proteins were performed as described in Materials and Methods. The ratios of WT:G83D are based upon active fractions as described in Materials and Methods.

G83D is deficient for DNA glycosylase activity but is able to bind to DNA with a Tg:A base pair *in vitro* and exhibits increased retention time at psoralen crosslinks [14, 18]. Therefore, the presence of G83D in a DNA glycosylase reaction with WT NEIL1 could result in decreased enzymatic activity if it bound to and shielded Tg:A base pairs from excision by WT NEIL1. We expressed and pulled down NEIL1 WT and G83D from HEK-293T cells as described [10], and performed DNA glycosylase assays with a DNA substrate containing a Tg:A base pair as described in the Methods section. We demonstrate that WT NEIL1 has significantly greater activity than the G83D protein in this assay, as shown in Figure 1A (compare 10:0 vs 0:10 ratios of WT to G83D NEIL1, respectively; *p*<0.0001) (See Supplementary Figure 1 for quantification of proteins). A mixture of 5:5 WT and G83D proteins has significantly less activity than WT protein alone (compare 5:5 vs 10:0; *p* ≤ 0.01). Similar results are observed in mixing experiments with purified NEIL1 protein (Figure 1B). In summary, our results suggest that G83D acts in a dominant or co-dominant manner to WT during an *in vitro* DNA glycosylase assay and that its presence can impact the overall DNA glycosylase activity in the reaction, at least against the Tg substrate.

### Replication stress is present in cells expressing NEIL1 G83D

It has been proposed that NEIL1 functions ahead of replication forks to remove replication blocking oxidative lesions, acting as a “cowcatcher” [10], using a form of long-patch BER. Because G83D interferes with the removal of Tg by WT NEIL1 in an *in vitro* glycosylase reaction, we reasoned that higher levels of replication stress would be present in cells expressing G83D than in WT cells. We first expressed either NEIL1 WT or G83D in MCF10A immortal but non-transformed breast epithelial cells using the pRVY-TET vector [20, 21]. Briefly, we subcloned either hemagglutinin (HA)-tagged WT or cDNA encoding the NEIL1 G83D variant into the pRVYTET vector, prepared retrovirus, and infected MCF10A human breast epithelial cells, which endogenously express WT NEIL1 DNA glycosylase. Tagging the proteins with the HA epitope enabled us to distinguish endogenous WT from exogenous protein. We selected independent pools of MCF10A cells exogenously expressing equivalent quantities of either NEIL1 WT or NEIL1 G83D (Supplementary Figure 2) as described [22] [20].

**Figure 2:**
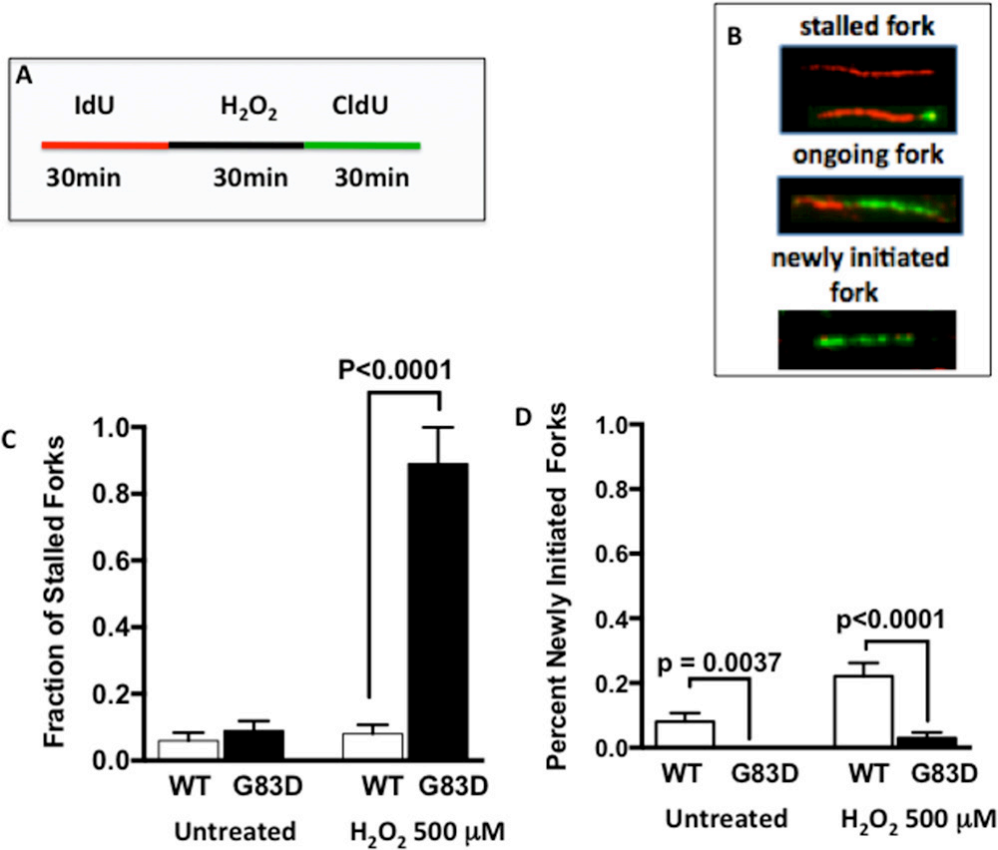
Expression of G83D NEIL1 induces replication stress We performed DNA fiber assays in the absence and presence of H_2_O_2_, which was used to induce oxidative base damage. **(A)** We first labeled the DNA of replicating cells for 30 minutes with IdU (red), and then treated or not with H_2_O_2_ to induce oxidative DNA damage. After treatment, we labeled the DNA with CIdU (green) to detect forks that continued to replicate. **(B)** Examples of images of stalled, ongoing, and newly initiated replication forks. **(C)** Quantification of spontaneous and H_2_O_2_-induced stalled forks. **(D)** Quantification of newly initiated forks. Unpaired t-tests were used to determine levels of significance.

**Figure 3:**
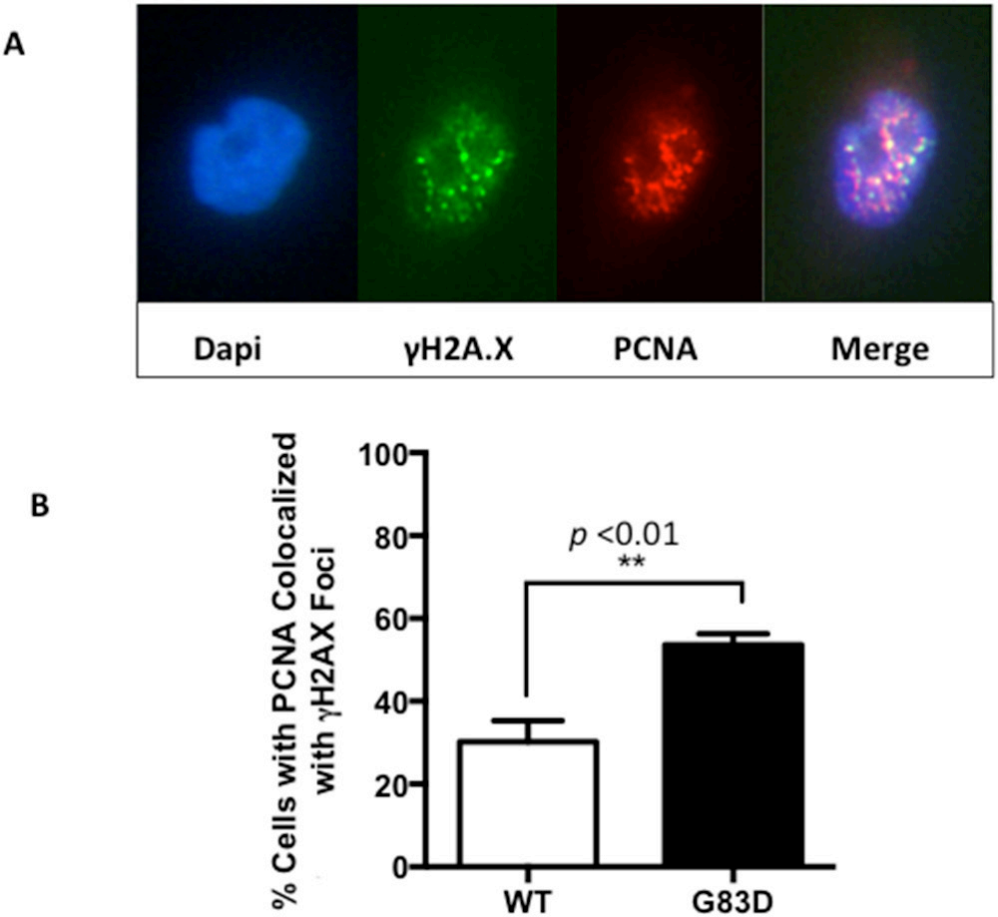
PCNA and γH2AX foci colocalize in cells expressing NEIL1 G83D Cells were treated with 0.2 mM H_2_O_2_ and analyzed as described in methods. Although colocalization of PCNA and γH2AX is observed in cells expressing WT NEIL1, greater levels of colocalization is observed in cells expressing G83D NEIL1. **(A)** Two examples of PCNA and γH2AX in the nucleus of a cell expressing G83D. **(B)** Quantification of cells exhibiting foci of PCNA that colocalize with γH2AX. Unpaired t-tests were used to determine levels of significance.

Next, to determine if replication stress is present at higher levels in G83D-expressing cells, we performed DNA fiber assays as we previously described [23] [24], in the absence and presence of H_2_O_2_, which was used to induce oxidative base damage. We first labeled the DNA of replicating cells for 30 minutes with IdU (red), and then treated with H_2_O_2_ to induce oxidative DNA damage (Figure 2A). After treatment, we labeled the DNA with CIdU (green) to detect forks that continued to replicate, most likely as a result of DNA repair. Our results show that cells expressing G83D have significantly increased levels of stalled replication forks when compared to cells expressing WT NEIL1 in the presence of oxidative DNA damage induced by H_2_O_2_ (Figure 2B and 2C). In addition, we observe significantly lower levels of newly initiated forks in cells expressing G83D versus WT NEIL1, both in the absence and presence of induced oxidative DNA damage (Figure 2B and 2D). Increased levels of stalled forks and decreased levels of newly initiated forks in G83D-expressing cells indicate that replication stress is present in these cells. Next, we quantified the numbers of γH2AX foci that colocalize with PCNA foci as another indicator of replication stress. Importantly, cells expressing G83D exhibit increased levels of colocalization of γH2AX and PCNA compared with WT cells, indicative of replication stress and perhaps the presence of DNA double-strand breaks (DSBs) at the replication fork (Figure 3).

**Figure 4:**
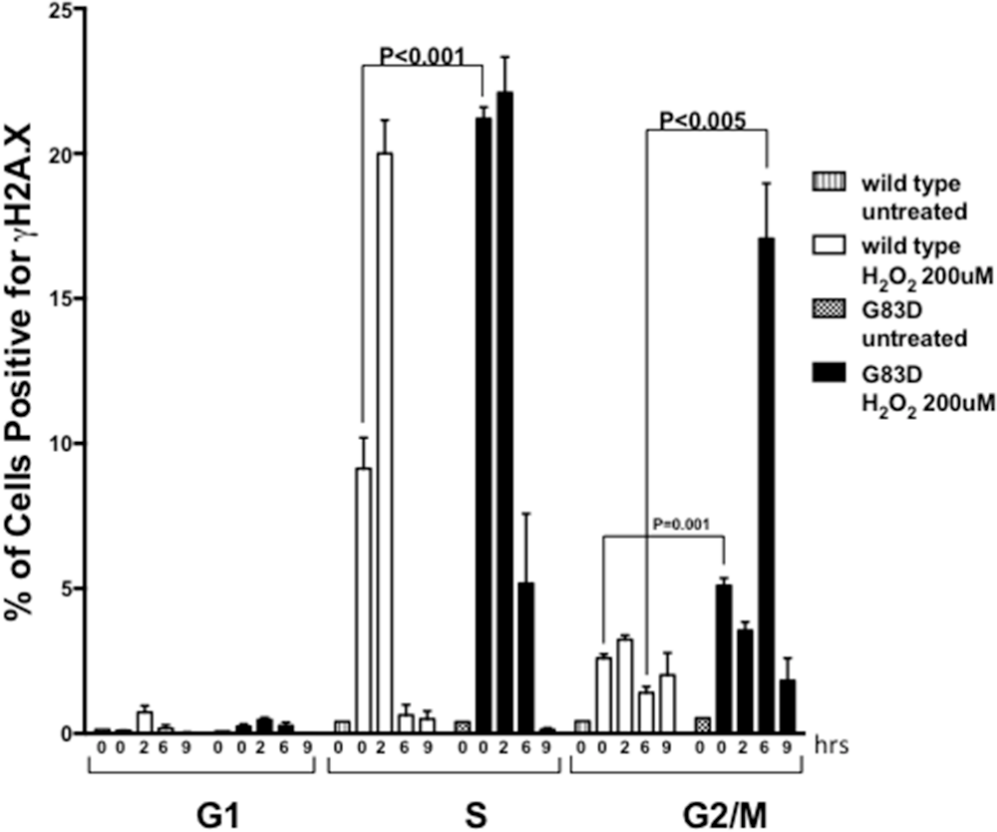
NEIL1 G83D induces DSBs during S and G2/M phases of the cell cycle Cells were synchronized as described in methods, released, and treated (or not) with 0.2 mM H_2_O_2_. Cells were fixed and stained with antisera against γH2AX/FITC to monitor DSBs, and with Propidium Iodide (PI) to monitor the phase of the cell cycle. Flow cytometry was then performed. Unpaired t-tests were used to determine levels of significance after various hours of recovery.

**Figure 5:**
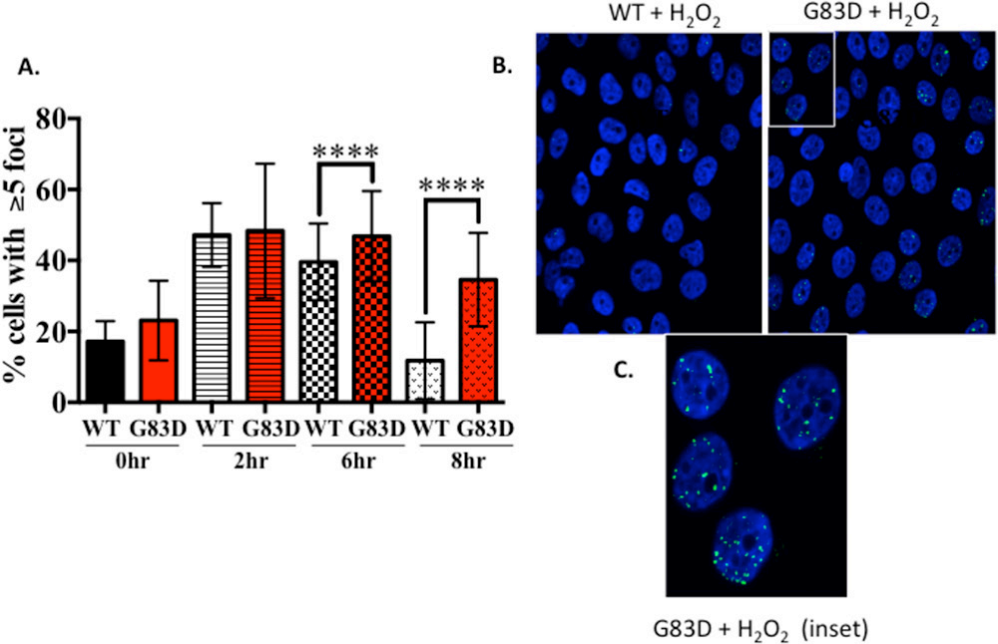
The levels of 53bp1 are higher in cells expressing G83D versus WT NEIL1 MCF10A pools expressing NEIL1 WT or G83D were treated 200μM H_2_O_2_ for 30m then fixed at 0, 2, 6 and 8hrs post-treatment and immunofluorescence was performed. Cells were labeled with a 53BP1 antibody (green) then mounted in Prolong Gold mounting media containing DAPI (blue, nuclei). Labeled cells were visualized using a Zeiss LSM 510 META confocal imaging system. **(A)** The number of nuclei with greater than 5 foci of 53BP1 was counted. The data are graphed as mean ± SD (n>500 nuclei) **** p< 0.0001. **(B)** Representative images of 53BP1 foci in MCF10A NEIL1 WT and G83D G151D expressing pools at 8hrs post-treatment. **(C)** Expanded inset of nuclei outlined in part B.

Next, we characterized the presence of DSBs as a function of cell cycle phase. We synchronized cells for 24 hours by serum and growth factor deprivation, followed by growth in complete medium for 18 hours to reach S phase (Supplementary Figure 3). We then treated the cells with H_2_O_2_ during S phase and quantified the levels of γH2AX as a function of the cell cycle phase. As shown in Figure 4, we observed significantly increased levels of γH2AX during both S and G2/M phases in cells expressing G83D versus WT immediately after treatment with H_2_O_2_. However, both G83D- and WT-expressing cells possessed similar levels of γH2AX in S phase after 2 hours of recovery from treatment with H_2_O_2_. After 6 hours of recovery from treatment, cells expressing G83D exhibited significantly increased levels of γH2AX compared to WT cells during the G2/M phase of the cell cycle. To provide additional evidence for the presence of DSBs in the cells expressing G83D, we stained cells with antisera raised against 53bp1. We demonstrate that there are greater levels of 53bp1 foci, marking DSBs, in cells expressing G83D versus WT NEIL1, at 6 and 8 hours after treatment with hydrogen peroxide (Figure 5). In combination, these results suggest that higher levels of DSBs arise in cells expressing G83D compared to WT NEIL1.

**Figure 6:**
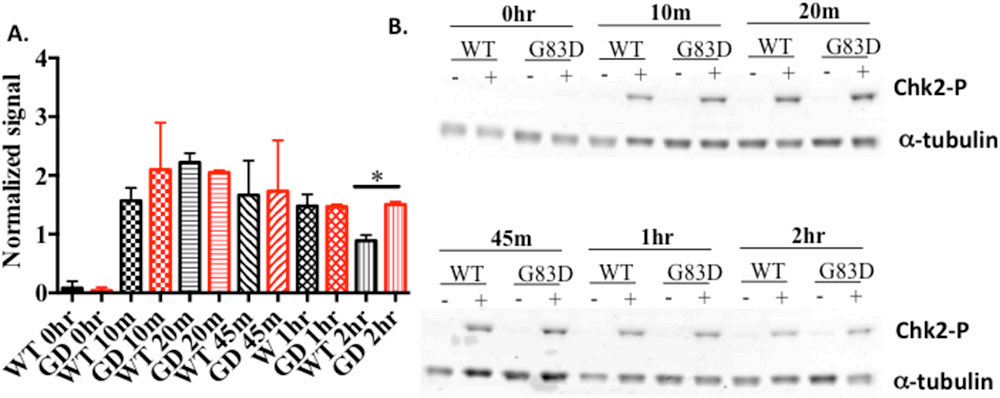
Chk2 is phosphorylated to greater levels in cells expressing G83D **(A)** Quantification of levels of phosphorylated Chk2, normalized to alpha tubulin, at various times after treatment with hydrogen peroxide. **(B)** Example of a western blot using anti-Chk2 (Ser 68).

Replication stress is also known to be associated with phosphorylation of the CHEK1 (Chk1) kinase and subsequent initiation of cell cycle checkpoints. We show that Chk1 is phosphorylated in both WT and G83D cells to similar levels after treatment with hydrogen peroxide (Supplementary Figure 4). However, we observe that phosphorylation of CHEK2 (Chk2) kinase is increased in cells expressing G83D after treatment with hydrogen peroxide (Figure 6), signifying the presence of DSBs.

### Expression of NEIL1G83D in MCF10A cells induces genomic instability and mutagenesis

**Figure 7:**
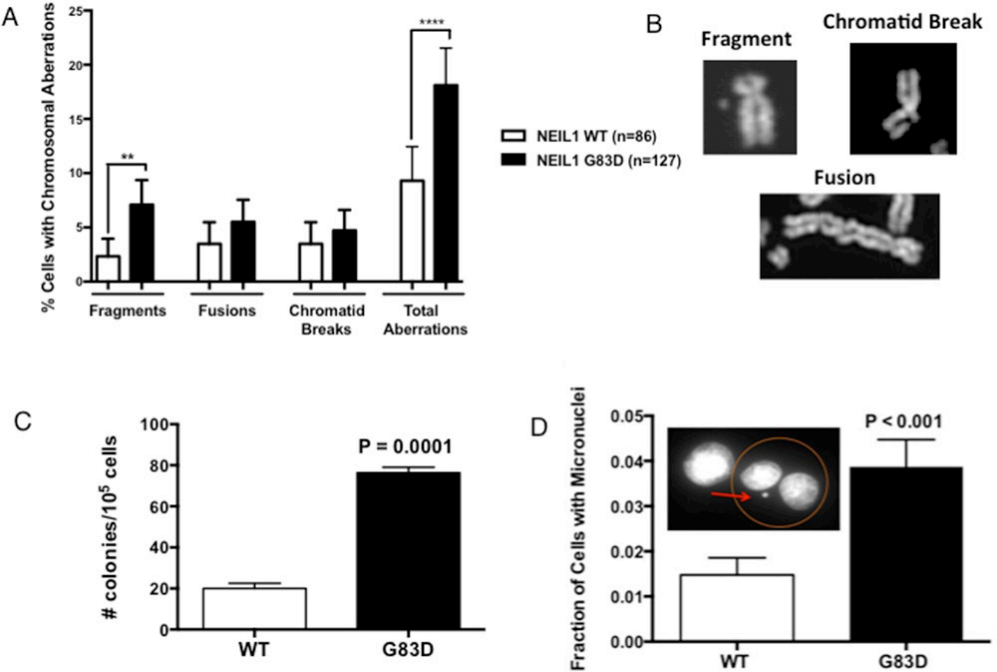
G83D induces genomic instability and mutagenesis **(A)** Assessment of chromosomal aberrations. Experiments were performed as described in methods using cells at passage 2. Expression of the G83D variant induces significantly increased numbers of fragments and total aberrations compared to WT. At least 50 nuclei were analyzed. ** p=0.007; **** p < 0.0001. **(B)** Examples of types of chromosomal aberrations scored in the assay. **(C)** Determination of mutagenic potential. Oubain resistance was scored in cells at passage 2 as described in methods. Expression of G83D induces point mutations at a significantly increased frequency over that of WT. **(D)** Measurement of micronucleus formation. Micronuclei were scored as described in methods. The G83D cells exhibit significantly increased levels of micronuclei when compared with WT NEIL1-expressing cells.

Replication stress is associated with genomic instability [25–27], especially arising during G2/M in the form of micronuclei. Because G83D NEIL1 acts in a dominant negative or co-dominant manner to WT in an *in vitro* DNA glycosylase assay and its expression induces replication stress, we reasoned that expression of NEIL1 G83D DNA glycosylase in the presence of WT NEIL1 would result in genomic instability and/or mutagenesis. To determine if expression of G83D induces genomic instability, we prepared and analyzed metaphase spreads of MCF10A cells alone or expressing either G83D or WT NEIL1. Expression of G83D leads to significantly increased levels of chromosomal fragments when compared to cells expressing WT NEIL1 as well as increased levels of total aberrations when compared to WT NEIL1 and MCF10A cells alone (Figure 7A and 7B). We also determined whether the presence of G83D results in an increased mutation frequency by assessing the ability of MCF10A cells expressing either G83D or WT NEIL1 to grow in medium containing ouabain. Ouabain binds to and inhibits the Na^+^/K^+^-ATPase sodium potassium ion pump, leading to accumulation of intracellular sodium and eventual cell death. Mutations in the Na^+^/K^+^-ATPase sodium potassium ion pump can result in ouabain resistance, therefore increased ouabain resistance serves as a marker for increased mutagenesis. As shown in Figure 7C, cells expressing NEIL1 G83D exhibited a significantly increased mutation frequency when compared to WT cells as monitored by ouabain resistance. Increased formation of micronuclei is also reflective of underlying replication stress and genomic instability. Interestingly, cells that express G83D exhibit significantly higher levels of micronuclei than WT-expressing cells, as shown in Figure 7D. Therefore, expression of G83D in MCF10A cells induces genomic instability and an increased mutation frequency when compared to cells expressing WT NEIL1.

### NEIL1 G83D induces cellular transformation

**Figure 8:**
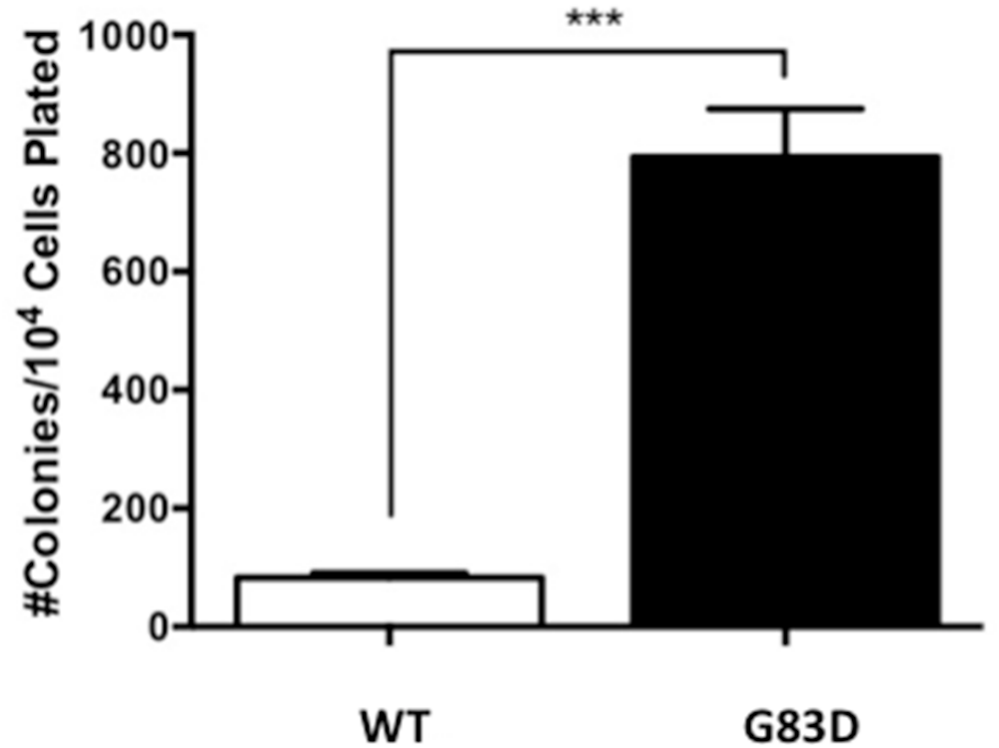
G83D expression induces anchorage-independent growth After selection of stable pools, the cells were passaged every 3-4 days and on every fourth passage plated in soft agar to assess anchorage-independent growth. Significantly greater numbers of colonies grow when cells express G83D NEIL1 versus WT at passage 12. Previous to this passage very few colonies growing in an anchorage-independent manner were observed.

Genomic instability, mutagenesis, and the presence of micronuclei are all linked to carcinogenic phenotypes. Therefore, we assessed the ability of G83D- versus WT-expressing cells to grow as colonies in soft agar, termed anchorage independent growth. We passaged the cells in the absence of dox and characterized anchorage independent growth. At passage 12, cells expressing G83D together with endogenous WT NEIL1 exhibit significantly increased levels of anchorage independent growth compared with cells expressing WT NEIL1 alone (Figure 8).

## DISCUSSION

The overall goal of this study was to determine if the *NEIL1* G83D germline variant has a functional phenotype that is related to cancer. We found that expression of this variant in the presence of the WT NEIL1 DNA glycosylase in human MCF10A immortalized breast epithelial cells leads to increased levels of replication stress resulting in genomic instability and an increased frequency of mutagenesis. Importantly, we also show that cells expressing G83D can grow in an anchorage-independent manner after several passages. These phenotypes are often associated with carcinogenesis and suggest that individuals who harbor the *NEIL1* G83D variant may be at increased risk for cancer.

### NEIL1 G83D is dominant to WT NEIL1 *in vitro*

Upon co-incubation of G83D and WT NEIL1, we show that G83D blocks or interferes with the removal of Tg, a replication-blocking lesion. Because G83D is able to bind to Tg but not remove it [14], we suggest that even in the presence of WT NEIL1, G83D binds to Tg, and shields it from excision by WT NEIL1. We observe this to be the case even when the concentration of G83D is less than half that of WT NEIL1. We suggest that G83D also binds to lesions, including Tg, arising in the DNA of cells, probably shielding the lesions from immediate excision by WT NEIL1 and perhaps other DNA glycosylases.

### The presence of G83D NEIL1 interferes with BER at the fork

Mitra and colleagues [10] proposed that a major role of NEIL1 DNA glycosylase is to act as a “cowcatcher” ahead of the replication fork. Specifically, it is proposed that NEIL1 binds to oxidative lesions in single-stranded DNA (ssDNA) proximal to the fork and that the presence of RPA inhibits excision of the damaged base and cutting of the DNA backbone, limiting DSB formation. It is suggested that the binding of NEIL1 results in replication fork reversal and once the fork is reversed, NEIL1 excises the damaged base, cuts the DNA backbone, and utilizes long patch BER involving an interaction with DNA polymerase δ (Pol δ) to complete DNA repair, allowing replication fork progression. In fact, depletion of NEIL1 significantly slows replication fork progression [10].

In cells expressing the glycosylase-deficient G83D enzyme, we observe a significant increase in the levels of stalled replication forks over what is seen in cells expressing WT NEIL1 in the presence of oxidative DNA damage induced by H_2_O_2_. We also observe increased levels of γH2AX in cells expressing exogenous G83D versus WT NEIL1 during S phase immediately after H_2_O_2_ treatment, and during G2/M six hours after treatment. The results are consistent with the suggestion that DSBs arise during replication and are likely to be present at replication forks at higher levels in the cells expressing G83D. Our results are also consistent with the idea that DSBs arising during S phase may not be repaired until the cells reach the G2/M phase of the cell cycle.

NEIL1 G83D is able to bind to Tg, a replication-blocking lesion. Binding of Tg and perhaps other replication blocking lesions by G83D may shield them from WT NEIL1 and perhaps other DNA glycosylases with overlapping substrate specificity. An inability to remove replication-blocking lesions would not promote repair by long patch BER, leading to irreversible stalling of the replication fork, perhaps prolonged fork regression and subsequent processing by nucleases, for example, MUS81 or the SLX4 complex [28] (for a review see [29]), leading to replication fork collapse. Interestingly, we observe increased levels of Chk2, but not Chk1, phosphorylation in cells expressing G83D. This is consistent with the presence of prolonged fork regression catalyzed by FBH1 helicase and subsequent MUS81-dependent processing of the regressed fork to generate DSBs, which triggers phosphorylation of Chk2 [30–32]. Alternatively, as G83D and WT NEIL1 are both present in the cells, G83D and WT NEIL1 may be in rapid exchange of binding to the replication-blocking lesion. This may alter replication fork dynamics in such a way that base excision occurs in a non-regressed fork, leading to induction of a DSB.

The DSBs that arise as a result of replication fork collapse or inappropriate base excision at the replication fork likely give rise to the chromosomal aberrations and micronuclei that we find at increased levels in cells expressing NEIL1 G83D. We suggest that these types of genomic instability arise and accumulate during passaging of the cells expressing NEIL1 G83D and together with selection for highly proliferative cells during passaging, eventually result in the ability of the cells to grow in an anchorage independent manner.

### Increased levels of mutagenesis may also promote anchorage independent growth

Anchorage of cells to the extracellular matrix (ECM) regulates both proliferation and cell division (for a review see [33]). Anchorage-independent growth results when cells are able to grow and divide in the absence of ECM, as a result of oncogenic mutations, with some of the most well known mutations occurring in the *RAS* oncogenes. In addition to chromosomal aberrations, we also observe significantly increased levels of point mutations arising in cells expressing NEIL1 G83D. We suggest that some of the oxidized bases that are not removed when G83D is present in the cells are bypassed by translesion DNA polymerases at the replication fork, resulting in mutations in a form of damage tolerance. Some of these mutations may arise in oncogenes or other genes that lead to circumvention of the requirement for the cells to be anchored during proliferation and cytokinesis, ultimately resulting in anchorage independent growth.

## SUMMARY

Our results demonstrate that expression of the NEIL1 G83D glycosylase-deficient enzyme in immortal but non-transformed human cells leads to the accumulation of stalled replication forks, increased levels of genomic instability and mutagenesis, resulting in cellular transformation. Our results are consistent with the suggestion that individuals who harbor the germline SNP encoding for NEIL1 G83D have increased levels of genomic instability that could predispose them to the development of cancer.

## MATERIALS AND METHODS

### Cell lines and cell culture

The MCF10A cells were grown as described previously [21]. The GP2-293 virus packaging cell line (Clontech) was used for retrovirus preparation. These cells were maintained in Dulbecco modified Eagle’s medium (Invitrogen) supplemented with 10% fetal bovine serum (Invitrogen), 1% L-glutamine (Invitrogen), 1% penicillin-streptomycin (Invitrogen) and 1 mMHEPES (Invitrogen). HEK293T cells were gown in DMEM with 110 mg/L sodium pyruvate, 6mM L-glutamine, 10% fetal bovine serum, and 100 U/mL penicillin-streptomycin.

### Cloning of NEIL1 WT and G83D and expression in MCF10A cells

The strategy used to subclone WT and G83D NEIL1 was similar to the one described previously for NTHL1 [21]. For cell culture experiments, human NEIL1 cDNA with a C-terminal hemagglutinin (HA) tag was cloned into the pRVYTet retroviral vector as described [20]. The G83D NEIL 1 variant was introduced into the human WT NEIL1cDNA sequence using site-directed mutagenesis (Stratagene) following the manufacturer’s protocols as described previously [14]. Human NEIL1 WT and G83D constructs were packaged into retrovirus using the GP2-293 packaging line as previously described [22]. Expression of exogenous HA-tagged NEIL1 was verified by Western blot using monoclonal mouse anti-HA antibody(Covance). β-tubulin(Sigma-Aldrich) was used as a loading control. pHCMV1 NEIL1 MBP-His was restricted with NotI and XhoI to remove the MBP-his sequence and replaced with a FLAG sequence to create pHCMV1-NEIL1 FLAG. The pHCMV1-NEIL1G83D-Flag variant plasmid was made using the Q5 Site-Directed Mutagenesis kit (New England Biolabs, NEB), following the NEB protocol with the following primers, CTCTTTTCAGCTGGTGCCCCGC forward and TCGGACATGCCGAAGCGGAAGACC reverse. The sequences were confirmed by the University of Vermont Advanced Genome Technology Core.

### Expression and pull-down of NEIL1 from HEK293T cells

HEK-293T cells were transiently transfected with the pHCMV1-NEIL1 FLAG or the pHCMV1-NEIL1G83D-Flag variant plasmids using the calcium phosphate method as described (delangelab.rockefeller.edu/assets/file/293T_Tx_co-IP.pdf). HEK-293T cells were harvested approximately 48 hours after transient transfection. Media was removed from a 100 mm plate and cells were scraped in 10 mls ice cold phosphate buffered saline (PBS), collected and centrifuged at 400 x G for 5 minutes at 4°C, the supernatant was aspirated. The cells were lysed in 1 ml of 50 mM Tris (pH 7.5), 150 mM NaCl, 1 mM EDTA, 1% Triton X-100 (SIGMA lysis buffer), 1 mM NaF, 1 mM Na_3_VO_4_, 1 mM PMSF and Roche Complete Ultra EDTA-free protease inhibitor (according to manufacturers instructions) for 30 minutes on ice. The lysate was centrifuged at 4°C for 15 minutes at 19090 x G, the supernatant was added to ANTI-FLAG M2 magnetic beads (20 μl, SIGMA) in 1.5 ml microcentrifuge tubes and incubated at 4°C for 2 hours with inversion. The microcentrifuge tubes were placed in a magnetic separator and the beads were washed twice with 500 μl of 50 mM Tris-Cl, pH 7.5 and 150 mM NaCl (tris buffered saline-TBS). The NEIL1 complexes were eluted with 100 μl of 100 μg/ml flag peptide in TBS and 10% glycerol at 4°C for 30 minutes with inversion. The M2 magnetic beads were removed and the complexes were assayed immediately or frozen at -80°C.

### DNA glycosylase assays

Glycosylase assays were carried out with 10 nM Tg:A substrate. The substrate was made by labeling 1 pmol of the Tg containing oligonucleotide with T4 polynucleotide kinase (New England Biolabs) and γ–^32^P for 15 minutes at 37°C. The reaction was stopped by the addition of 1 mM EDTA and heating to 95°C for 1 minute. The DNA was ethanol precipitated and dried, 9 pmol of the Tg containing oligonucleotide was added along with 10 pmol of the complementary oligonucleotide in 10 mM Tris (pH 8.0) and 50 mM NaCl at a final concentration of 250 nM double-stranded oligonucleotide substrate. The substrate was placed in a boiling water bath and cooled slowly to allow the DNA to anneal. The oligonucleotide sequences were as follows: the damage containing strand 5’-TGTCAATAGCAAGTgGGAGAAGTCAATCGTGAGTCT-3’, the complementary strand 5’-AGACTCACGATTGACTTCTCCACTTGCTATTGACA-3’.

Glycosylase/lyase assays contained a final concentration of 10 nM Tg:A substrate, 25 mM Tris (pH 7.5), 75 mM NaCl, 10 mM MgCl_2_, 200 μg/mL bovine serum albumin (BSA), 1 mM dithiothreitol (DTT) and NEIL1 immunoprecipitation elution. The assays were allowed to proceed for 60 minutes and quenched in an equal volume of formamide stop solution (98% formamide, 0.1 mM EDTA. 0.1% bromophenol blue and 0.1% xylene cyanol). The samples were resolved on a 12% polyacrylamide (w/v) sequencing gel, transferred to Whatman 3M paper, dried and exposed to a phosphor imager screen and imaged with a Pharos FX Plus Molecular Imaging System (BioRad).

Glycosylase/lyase assays with purified his-tagged NEIL1 WT and G83D were performed using the same conditions as used in the immunoprecipitation assay except that the concentration of Tg:A substrate was increased from 10 to 200 nM. A Schiff base assay was performed to determine the active fraction of NEIL WT and G83D [34].

### Western blotting

To perform quantitative western blotting for NEIL1, eluted NEIL1 complexes from the FLAG tag pull down were resolved in a 10% SDS-page gel along with 100, 200, 400, 600 and 800 ng of His-tagged recombinant NEIL1 purified from *E. coli* as described previously [14]. The proteins were transferred to an Immobilon-FL PVDF membrane (EMD Millipore) blocked for 1 hour and probed with a rabbit polyclonal antibody to NEIL1 (Abcam), (5/10,000) for 1 hour. The membrane was washed and incubated with IRDye 680RD goat anti-rabbit IgG (1/20,000) for 30 minutes. All incubations were performed at room temperature. The membrane was washed and visualized on a LiCor Odyssey CLx Infrared Imaging System. The expression levels of NEIL1 were quantified using Image Studio Software version 2.1.10. A linear regression was performed using the known amounts of the His-tagged NEIL1 and the amounts of NEIL1 WT and G83D were determined.

### DNA fiber assay

This assay was performed as described [24]. Briefly, MCF10A cells expressing WT or G83D NEIL1 were grown to approximately 30-40% confluence. Cells were pulsed with 25 μM IdU for 30 minutes, washed three times with PBS, and pulsed with 250 μM CldU for 30 minutes. Cells were treated or not with 500 uM H_2_O_2_ for 30 minutes on ice between pulses. Cells were harvested and resuspended in PBS at a concentration of 1.7 x 10^6^ cells/ml and 3 μl (5000 cells) of the cell suspensions were placed on glass slides and mixed with 7 μl of lysis buffer (200 mM Tris, pH 7.6, 50 mM EDTA, 0.5%SDS) for 2 min. Slides were tilted at 20° for gravity flow. The slides were fixed in a 3:1 solution of methanol-acetic acid for 20 min at -20°C and treated with 2.5 M HCL for 30 min followed by washes with PBS before blocking in 5% BSA for 30 min at 37°C. To detect incorporated IdU and CldU, DNA fibers were incubated with mouse anti-BrdU (Becton Dikinson; 1:25) and rat anti-BrdU monoclonal antibody (Abcam; 1:400), respectively, for 1 h at room temperature (RT), followed by 3 washes with PBS, and incubation with sheep anti-mouse Cy3 (Sigma; 1:500) and goat anti-rat Alex Fluor 488 (Invitrogen 1:400) for 1 h at RT. The slides were mounted with Vectashield mounting medium and covered with coverslips. Images were acquired using a Zeiss microscope at 63 magnification and processed using Image J software (http://imagej.nih.gov/ij/).

### Anchorage-independent growth assay

Anchorage-independent growth was assessed as previously described [20]. Briefly, 1 x 10^4^ MCF10A cells expressing either WT or G83D NEIL1 were mixed with media containing 0.7% noble agar (USB) and poured onto a layer of media containing 1.0% noble agar. The number of colonies present in each of ten microscope fields per well from a total of 3 wells per experiment were counted after 4 weeks of growth.

### Immunofluorescence

MCF10A cells expressing WT or G83D NEIL1 were grown on glass coverslips (Sigma). Cells were fixed in a 3:1 solution of methanol-acetic acid for 20 min at -20°C and permeabilized in 0.5% Triton buffer (20 mM HEPES, pH 7.4, 50 mM NaCl, 3 mM MgCl_2_, 300 mM sucrose, 0.5% Triton x-100) for 10 min at RT. Coverslips were blocked in 3% BSA and goat serum for 30 min at RT and incubated with the primary antibodies, anti-PCNA (Santa Cruz) or anti-γH2AX (Cell Signaling) overnight at 4°C. Coverslips were washed and incubated with secondary FITC or rhodamine antibodies. Coverslips were mounted on slides using SlowFade^®^ Gold Antifade Mountant containing DAPI to stain the nuclei (Invitrogen). To determine if 53bp1 foci were present after induction of oxidative damage, cells were treated 0 or 200μM H_2_O_2_ then allowed to recover for 0, 2, 6 or 8hrs post-treatment. Cells were washed 2 times with PBS then fixed (4% paraformaldehyde, 0.02% TritonX-100) for 15 minutes at room temperature. Cells were rinsed with PBS then incubated with blocking/permeabilization solution (10% normal goat serum, 0.5% TritonX-100) for 1 hour at with gentle shaking. The blocking/permeabilization solution was then replaced with blocking/permeabilization solution containing diluted (1/500) mouse anti-human 53BP1 primary antibody (Millipore; MAB3802) and incubated overnight at 4°C with gentle shaking. The next day, the cells were washed with PBS/0.5% TritonX-100 followed by 2 washes with PBS. Cells were then incubated with AlexaFluor 488 goat anti-mouse IgG (H+L) antibody (1/1000) (Invitrogen) diluted in blocking/permeabilization solution for 1 hour with gentle shaking. The cells were washed with PBS/0.5% TritonX-100 followed by 2 washes with PBS then slides were mounted in Prolong Gold Antifade reagent with DAPI (Invitrogen). Cells were imaged using a Zeiss LSM 510 META confocal scanning laser microscope.

### Flow assay and western blotting for Chk1 and 2

This assay was conducted essentially as described [21]. Briefly, MCF10A cells expressing WT or G83D NEIL1 were plated and synchronized by serum starvation for 48 hours, then complete media was added back and cells allowed to recover for 18 hours until entering S-phase. Cells were then treated with 200 μM H_2_O_2_ on ice for 20 minutes, placed back into media and allowed to recover for the specified times. Cells were harvested on ice in cold Accutase with 5 mM EDTA, phosphatase and protease inhibitors, washed and fixed in ice cold 70% EtOH and stored at -20°C for at least 30 min and up to a month. For flow cytometry, cells were rehydrated and resuspended in PBS + 1% BSA, 0.1% Triton X-100, 5mM EDTA, 3X phosphatase inhibitors and 1X protease inhibitors, then stained with anti-histone H2A.X antibody - ChIP Grade (ab11175) overnight at 4^°^C, and then with FITC-conjugated anti-rabbit secondary antibody (Alexa 488, BD Biosciences) for 1 hr at room temperature, all in the same buffer. They were then washed and resuspended in 450ul BD PI/Rnase buffer with 5 mM EDTA, 3X phosphatase inhibitors and 1X protease inhibitors and strained through 44 micron filter mesh into a flow cytometry tube. Fluorescence was analyzed by flow cytometry using the BD FACSCalibur and analyzed using FlowJo 8.8.6 software. To monitor phosphorylation of Chk1 and 2, samples of cells were also taken at various timepoints as shown in Figures 6 and Supplementary Figure 4, lysed and immunoblotted as described [21], with rabbit polyclonal phospho-Chk2 (Thr68) (Cell Signaling, catalog # 2661). Chk1 was immunoblotted with rabbit monoclonal phospho-Chk1 (Ser345) (Cell Signaling, cat # 2348). Chk1 and 2 levels were normalized against mouse monoclonal alpha tubulin, (Abcam, ab80779, clone DM1A).

### Genomic instability analysis

Chromosomal aberrations were assessed as previously described [22]. Cells were harvested by mitotic shake-off and lysed in 0.75% KCl at 37°C for 30 minutes before fixing in Carnoy’s Fixative (75% methanol, 25% acetic acid). Images were taken using Spot Camera software (Diagnostic Instruments). Metaphase spreads were de-identified and scored by eye for chromosomal fusions, breaks, and fragments. Asynchronous MCF10A cells were treated with ionizing radiation or 200 μM H_2_O_2_ on ice for 20 mins while in exponential growth. Fresh media containing 6 μg/ml Cytochalasin-B was added directly after exposure, followed by 24 hr incubation at 37°C. Cells were then trypsinized, washed, and resuspended in 7 ml 0.075 M (0.56%) potassium chloride for 10 min at 37°C, and fixed by adding 3 ml of 100% methanol for at least 1hr at room temperature, followed by fixation twice in acetic acid/methanol (1:3). Fixed cells were dropped onto wet slides, dried and stained with 10 μg/ml Acridine Orange (AO) in PBS for 20 min, and rinse briefly in water. A coverslip was placed over the cells in a drop of water on the slide and scoring was done immediately on an Olympus BX50 fluorescence microscope with a FITC (488 emission) filter cube. More water was added to the edge of the coverslip to prevent drying out during scoring. The slides were coded and scored blindly. For each sample, micronucleus induction in 100 binucleated cells was scored following the criteria described in detail in Fenech [35].

### Ouabain mutagenesis assay

MCF10A cells expressing WT or G83D NEIL1 in exponential growth. Cells were trypsinized and plated in 10 cm dishes at various concentrations. Cells were allowed to attach overnight and ouabain (Sigma) was added to a final concentration of 100 nM. After 3 weeks of growth, cells were stained with 0.25% crystal violet (Sigma). Colonies were counted and mutagenesis was calculated by dividing the total number of ouabain resistance colonies by the number of surviving colonies on plates grown in the absence of ouabain.

## SUPPLEMENTARY MATERIALS FIGURES



## Abbreviations

PI: Propidium Iodide
HA: Hemagglutinin tag
BER: Base excision repair
WT: wild-type
DSBs: double-strand breaks
RONs: reactive oxygen and nitrogen species
APE I: apurinic/apyriminidinic endonuclease I
pol ß: DNA polymerase beta
LIG3α: DNA ligase 3α
XRCC1: X-Ray Cross-Complimenting 1
dRP: deoxyribose phosphate
G83D: Gly83 to Asp83
Sp: spiroiminohydantoin
Gh: guanidinohydantoin
Tg: thymine glycol
ssDNA: single-stranded DNA
Pol δ: DNA polymerase δ
PBS: phosphate buffered saline
TBS: tris buffered saline
BSA: bovine serum albumin
DTT: dithiothreitol
AO: Acridine Orange
SNP: single nucleotide polymorphism
