# Supplementary material for: The NEIL1 G83D germline DNA glycosylase variant induces genomic instability and cellular transformation
Source: Oncotarget. 2017 Sep 8;8(49):85883–95. doi: 10.18632/oncotarget.20716 (PMC5689654; doi:10.18632/oncotarget.20716)
Supplement: Supplementary file 1 [file oncotarget-08-85883-s001.pdf]

The NEIL1 G83D germline DNA glycosylase variant induces genomic instability and cellular transformation

SUPPLEMENTARY MATERIALS

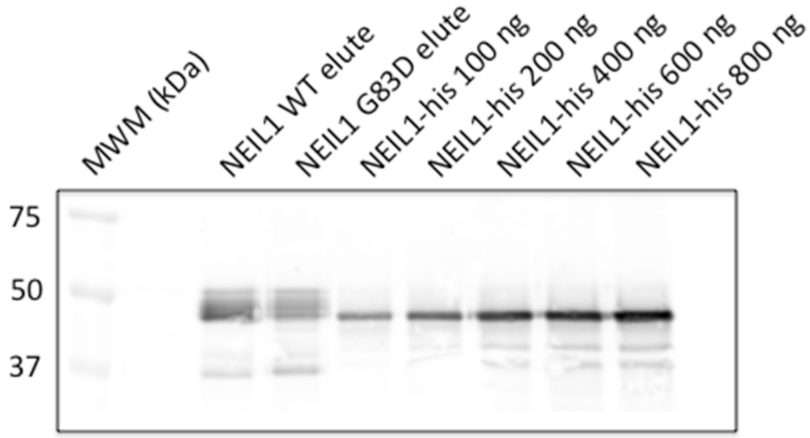

Supplementary Figure 1: Quantitative western blotting of NEIL1 protein fractions. Eluted NEIL1 complexes from the FLAG tag pull down, as described in the material and methods, were resolved in an SDS-page gel along with known amounts of His-tagged NEIL1. The proteins were transferred to a PVDF membrane and probed with a polyclonal antibody to NEIL1. The bands corresponding to NEIL1 were quantified and a linear regression was performed using the known amounts of the His-tagged NEIL1. The amount of NEIL1 WT and G83D were determined using a linear regression equation.

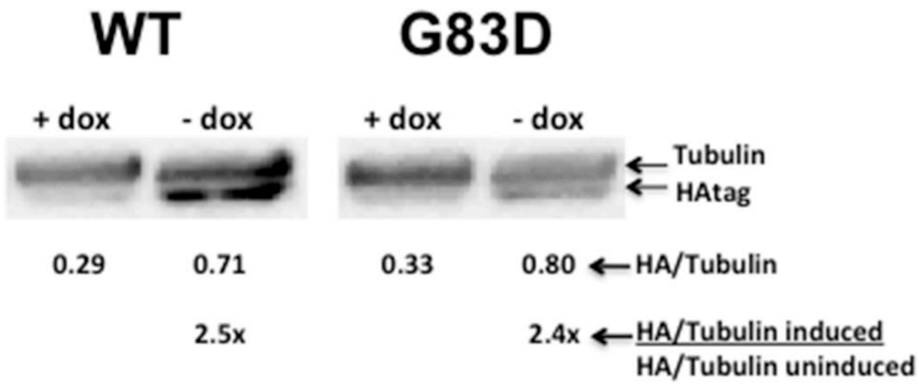

Supplementary Figure 2: Western blot of MCF10A cells expressing either WT or G83D NEIL1. Cells were harvested in RIPA buffer, lysed, and resolved on an SDS-PAGE gel. After blotting to PVDF the membrane was probed with antisera raised against the Hemagglutinin tag (HA) or tubulin, used as a loading control. In the presence of Dox, little expression of HA-tagged NEIL1 is present, but in the absence of Dox, NEIL1 is expressed. Expression of NEIL1 was normalized to that of Tubulin. The ratio of the band intensities of HA/tubulin is 0.71 for WT NEIL1 and 0.80 for G83D NEIL1. Therefore, equivalent expression is observed.

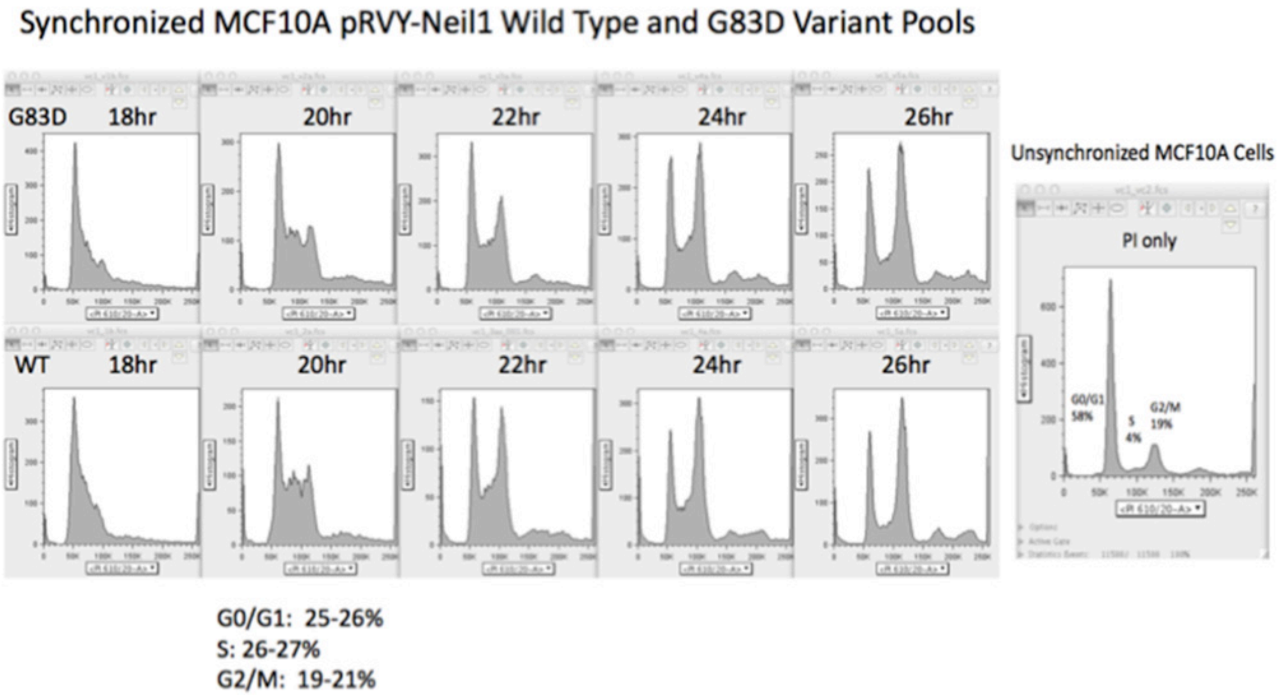

Supplementary Figure 3: Cell cycle profiles of cells expressing exogenous G83D or WT NEIL1. We synchronized cells for 48 hours by serum and growth factor deprivation, followed by growth in complete medium for 18 hours to reach S phase. We then performed flow analysis with Propidium Iodide (PI) to profile the cell cycle phases as a function of continued growth.

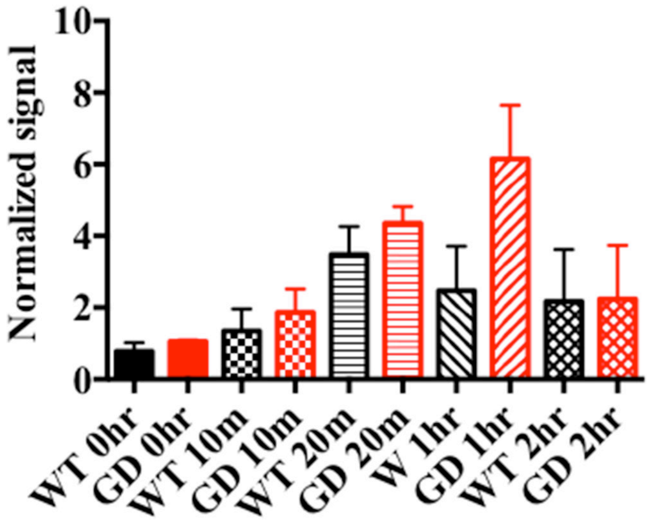

Supplementary Figure 4: Chk1 is phosphorylated at similar levels in cells expressing G83D and WT NEIL1. Quantification of levels of phosphorylated Chk1, normalized to alpha tubulin, at various times after treatment with hydrogen peroxide.
